# Beneficial and detrimental genes in the cellular response to replication arrest

**DOI:** 10.1371/journal.pgen.1010564

**Published:** 2022-12-27

**Authors:** Luciane Schons-Fonseca, Milena D. Lazova, Janet L. Smith, Mary E. Anderson, Alan D. Grossman

**Affiliations:** Department of Biology Massachusetts Institute of Technology Cambridge, Massachusetts, United States of America; University of Wisconsin-Madison, UNITED STATES

## Abstract

DNA replication is essential for all living organisms. Several events can disrupt replication, including DNA damage (e.g., pyrimidine dimers, crosslinking) and so-called “roadblocks” (e.g., DNA-binding proteins or transcription). Bacteria have several well-characterized mechanisms for repairing damaged DNA and then restoring functional replication forks. However, little is known about the repair of stalled or arrested replication forks in the absence of chemical alterations to DNA. Using a library of random transposon insertions in *Bacillus subtilis*, we identified 35 genes that affect the ability of cells to survive exposure to an inhibitor that arrests replication elongation, but does not cause chemical alteration of the DNA. Genes identified include those involved in iron-sulfur homeostasis, cell envelope biogenesis, and DNA repair and recombination. In *B*. *subtilis*, and many bacteria, two nucleases (AddAB and RecJ) are involved in early steps in repairing replication forks arrested by chemical damage to DNA and loss of either nuclease causes increased sensitivity to DNA damaging agents. These nucleases resect DNA ends, leading to assembly of the recombinase RecA onto the single-stranded DNA. Notably, we found that disruption of *recJ* increased survival of cells following replication arrest, indicating that in the absence of chemical damage to DNA, RecJ is detrimental to survival. In contrast, and as expected, disruption of *addA* decreased survival of cells following replication arrest, indicating that AddA promotes survival. The different phenotypes of *addA* and *recJ* mutants appeared to be due to differences in assembly of RecA onto DNA. RecJ appeared to promote too much assembly of RecA filaments. Our results indicate that in the absence of chemical damage to DNA, RecA is dispensable for cells to survive replication arrest and that the stable RecA nucleofilaments favored by the RecJ pathway may lead to cell death by preventing proper processing of the arrested replication fork.

## Author summary

DNA replication is essential for life. A variety of events, including chemical damage to DNA (e.g., caused by irradiation with ultraviolet light) and other cellular stresses that do not cause chemical modification of the DNA, can perturb and stop ongoing replication. Cell survival often depends on the ability to resume (restart) replication after such disruptions and many organisms have multiple mechanisms for restarting replication. Many bacterial species have two pathways for restarting DNA replication following DNA damage (e.g., exposure to ultraviolet light), and loss of either of these pathways makes cells more sensitive to UV irradiation. We found that one of these two pathways is detrimental and the other pathway is beneficial for bacterial survival following replication arrest in the absence of chemical damage to DNA. These surprising findings highlight key differences between the two pathways and the fine balance organisms face in maintaining replication restart pathways that are sometimes helpful and other times harmful.

## Introduction

DNA replication is essential for all living organisms, and a variety of events can perturb replication, including DNA damage and replication arrest due to “roadblocks” such as DNA-binding proteins or transcription. In any of these cases, some or all the components of the replication machinery (the replisome) may disassemble from the complex, leading to the collapse of the replication fork [1–3]. If a subsequent replisome reaches unrepaired lesions, nicks, or collapsed forks, it can turn these defects into double-strand breaks, which, if not repaired, are lethal.

Proper cell growth and division following replication arrest depend on the restart of replication. A wide range of bacteria use a similar mechanism for replication restart: the DNA is processed to enable the formation of a structure resembling a replication fork, followed by reassembly of the replisome (reviewed in [4]). This processing typically involves homologous recombination mediated by RecA [5, 6]. To initiate homologous recombination, helicases and nucleases process DNA at the arrested fork to generate single-stranded DNA (ssDNA), in a process called end-resection [7, 8].

*Bacillus subtilis* has two nucleases capable of performing end-resection, RecJ and AddAB. In response to DNA damage, the exonuclease activity of RecJ can expand ssDNA gaps by several kilobases [7, 9]. In contrast, the helicase-nuclease complex AddAB, a functional homolog of RecBC in *Escherichia coli*, binds blunt or near blunt DNA ends. AddAB degrades both strands until reaching a Chi site [10] where it switches to degrade only a single DNA strand in the 5’ to 3’ direction [8]. Both the RecJ and AddAB end-resection pathways in *B*. *subtilis* lead to loading of RecA onto the DNA by the loader protein RecO [11]. RecA forms nucleofilaments that can search and invade complementary double-stranded DNA, creating cross-shaped DNA structures known as Holliday junctions. Finally, Holliday junctions are processed into a substrate for the helicase and replication restart protein PriA. PriA functions downstream of AddAB and RecJ, binds to stalled replication forks and promotes assembly of the replicative helicase and primosome enabling replication restart (reviewed in [12, 13]).

Most of what is known about the bacterial response to replication arrest comes from experiments using DNA damage to disrupt DNA replication. Repair of damaged DNA and replication restart are both required for optimal survival, making it difficult to differentiate between processes related to the damage itself or the arrest of replication forks. We were interested in identifying genes that influence the ability of cells to survive replication arrest, without the confounding effects of chemical damage (e.g., pyrimidine dimers, cross-links) to DNA.

To cause replication fork arrest independent of DNA damage, we used HPUra (6-(p-hydroxyphenylazo)-uracil), an azopyrimidine that reversibly binds to and inhibits the catalytic subunit of DNA polymerase III (PolC) in *B*. *subtilis* [14]. We used Tn-seq (transposon insertion mutagenesis with massively parallel sequencing) to identify candidate genes that affect cell survival following the arrest of replication elongation caused by treatment with HPUra.

We found that many processes appeared to be important for survival, including: regulation of oxidative homeostasis, cell wall stability, and DNA repair pathways. Interestingly, we found that one of the DNA end-resection pathways that is important for surviving DNA damage was detrimental to surviving replication arrest. Loss of *recJ*, *recO*, and *recF* was beneficial to cell survival, indicating that the functional genes were detrimental. In contrast, functional *addA*, *recN*, and *recU* were beneficial for cell survival following replication fork arrest. Our findings indicate that although AddAB and RecJ both carry out end-resection, they commit replication fork repair to distinct pathways. We found that these pathways led to different amounts of RecA that formed filaments on the ssDNA. Following replication fork arrest, RecJ or the recombinase loader and regulator (*recO* and *recF*, respectively) led to excessive RecA loading, accumulation of unrepaired forks, and ultimately an increase in cell death. Our data indicate that *B*. *subtilis* activates at least two pathways to repair arrested replication forks due to replisome malfunction and that one pathway (AddAB) is beneficial and the other (RecJ) is detrimental to cell survival.

## Results

### Identification of genes involved in cell survival following replication arrest

Using Tn-seq, we identified non-essential genes that significantly affected the ability of cells to survive and recover from replication arrest caused by treatment with HPUra. We used a previously described library of ~1.7 x 10^5^ unique transposon insertions of a modified version of the *magellan6* transposon, *magellan6x*, distributed throughout the *B*. *subtilis* genome [15]. This library of cells was split in two and treated or not with HPUra for one hour. The cells were then removed from HPUra, allowing replication and cell growth to resume. Aliquots of cells were harvested 2, 3, and 4 hours later, and the location and relative number of transposon insertions in a given chromosomal site were determined by high-throughput sequencing. In a population of random transposon insertions, mutations that lead to decreased survival would be underrepresented, and the ratio between the frequency of insertions in treated and untreated samples would be <1. Conversely, insertion mutations that improved survival would be overrepresented, resulting in frequency ratios >1. The vast majority of genes had a similar number of insertions (frequency ratios of ~1) with or without HPUra, indicating that these are neutral (S1 Table).

**Fig 1:**
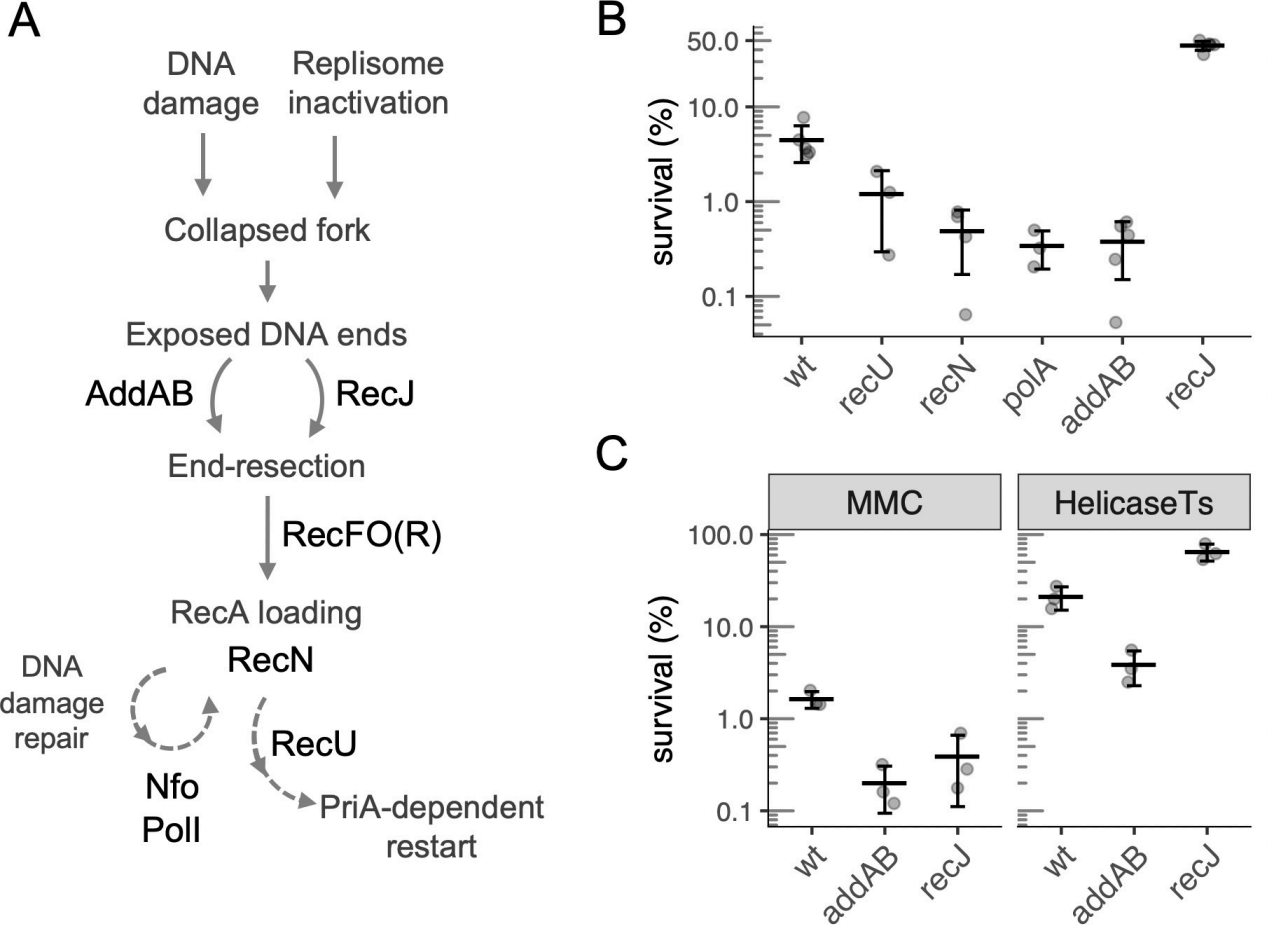
Pathways and genes involved in cell survival after arrest of DNA replication. **A.** General view of the roles of the DNA repair and recombination genes identified in the Tn-seq screen. The dashed arrows represent multistep pathways. **B.** Percent survival of wild type (strain JMA222) and *recU* (GP891), *polA* (LSF41), *recN* (LSF298), *addAB* (LSF253) and *recJ* (LSF200) null mutants after 3 h of replication arrest caused by HPUra treatment. Percent survival is relative to cell viability at the time of arrest. All mutants were statistically different from wild type, as assessed by two-sample t-tests (P < 0.025). **C.** Effects of *recJ* and *addAB* null mutations on cell survival following replication arrest due to treatment with the DNA damaging agent mitomycin-C (MMC) or inactivation of the replicative helicase (DnaC). Left panel: survival of wild-type (JMA222), Δ*addAB* (LSF253) and Δ*recJ* (LSF200) cells after 3 h of treatment with 0.33 μg/ml of MMC. Right panel: wild-type, Δ*recJ* and Δ*addAB* cells carrying a *dnaCts* allele (LSF176, LSF274, and LSF326) were grown at permissive temperature (30°C) to early exponential phase and shifted to non-permissive temperature (49°C) for 4 h. Percent survival is relative to the population before treatment (left) or the temperature shift (right). The deletion mutants in both conditions were statistically different from wild type, as assessed by two-sample t-tests (P < 0.025). Throughout this work, error bars correspond to one standard deviation of the mean and each point is from an independent culture (biological replicates).

Thirty-five genes were classified as affecting survival and recovery from HPUra (see Material and Methods for analysis and criteria), and 13 of these were validated using targeted gene disruptions (Table 1). Several cellular processes appeared to affect survival, including: (i) iron-sulfur and oxidative homeostasis (*rex*, *nadABC*, *defB*, and *nifS*), likely related to the induction of oxidative stress and genes regulating NAD/NADH following treatment with HPUra [16]; (ii) cell wall stability (*ltaS*, *rseP*, *oppAB*, *ydiL*, *cwlO* and *ponA*); (iii) induction of lysogenic phage (*yoyI*, *yonH* from the SPß prophage and *xpf*, *xkdBC* from PBSX); and (iv) DNA repair and recombination. Below, we focus on several of the DNA recombination and repair genes (Fig 1A). We also validated seven of the other genes identified in the screen using targeted gene disruptions (Table 1), but did not further evaluate them as part of this study. The number of insertion sites and read frequencies for all open reading frames are presented (S1 Table).

**Table 1:** Genes identified in Tn-seq analysis of cells surviving replication arrest in the absence of DNA damage.

Gene	Function	Log2 FC, Tn-seq^a^	Survival of null mutants^b^	Strain^b^
***DNA repair and recombination***				
*polA*	DNA polymerase I	-6.51	0.06 (0.01)	LSF41
*recU*	Holliday junction-specific endonuclease	-2.46	0.22 (0.09)	GP891
*recN*	recognition of DNA double strand breaks	-1.65	0.14 (0.08)	LSF298
*addA*	ATP-dependent deoxyribonuclease subunit A	-1.55	0.10 (0.06)	LSF253
*nfo*	endonuclease IV	-1.11	ND	
*recJ*	5’-3’ exonuclease	1.04	12 (1.7)	LSF200
*walJ*	metallo-β-lactamase/ 5’-3’ exonuclease	1.29	2.89 (0.07)	BKE40370
***Prophages***				
*yonH*	unknown function (SPß)	1.09	1.6 (0.3)^c^	LSF203
*yoyI*	transmembrane protein (SPß)	1.24		
*xkdC*	unknown function (PBSX)	1.39	1.9 (0.1)^d^	LSF204
*xkdB*	protein with LexA-type HTH domain (PBSX)	1.46		
*xpf*	positive control sigma-like factor (PBSX)	1.47		
***Iron-Sulfur and oxidative homeostasis***				
*rex*	redox-sensing transcriptional repressor	-1.24	ND	
*nadC*	nicotinate-nucleotide pyrophosphorylase	1.39	ND	
*nadA*	quinolinate synthetase	1.47	ND	
*defB*	peptide deformylase	1.64	ND	
*nifS*	cysteine desulfurase	1.84	ND	
*nadB*	L-aspartate oxidase	2.03	ND	
***Cell wall and membrane***				
*ltaS*	polyglycerolphosphate lipoteichoic acid synthase	-1.79	ND	
*rseP*	inner membrane zinc metalloprotease	-1.68	ND	
*oppA*	oligopeptide ABC transporter binding lipoprotein	-1.67	0.21 (0.07)	JRL131
*ponA*	peptidoglycan glycosyltransferase	-1.43	0.11 (0.03)	CMJ293
*oppB*	oligopeptide ABC transporter permease	-1.40	0.3 (0.11)	JRL189
*ydiL*	membrane protease	-1.05	ND	
*cwlO*	secreted cell wall DL-endopeptidase	1.10	2.2 (0.4)	CMJ374
***Other***				
*hrcA*	heat-inducible transcription repressor	-1.93	0.26 (0.09)	LSF233
*bkdB*	E2 subunit, lipoamide acyltransferase	-1.43	ND	
*speE*	spermidine synthase	-1.38	0.3 (0.15)	LSF20
*yutD*	unknown function	-1.12	ND	
*ytpQ*	unknown function	-1.10	ND	
*argG*	argininosuccinate synthase	1.07	ND	
*ywdH*	aldehyde dehydrogenase	1.08	ND	
*yoaQ*	unknown function	1.25	ND	
*ycgE*	transcriptional regulator	1.39	4 (2)	LSF444
*argH*	argininosuccinate lyase	1.42	ND	

^a^ Log2 FC: Log2 of the ratio of normalized number of insertions from the Tn-seq analyses in the indicated gene 4 h after removal of HPUra compared to that of cells that had not been treated with HPUra.

^b^ Survival after HPUra treatment of defined null mutants (strain numbers listed) relative to that of the isogenic parental strain, with one standard deviation of the mean in parenthesis.

^c^ Sensitivity of a strain devoid of SPß was used, rather than individual *yonH* and *yoyI* mutants.

^d^ Sensitivity of a strain devoid of PBSX was used, rather than individual *xkdC*, *xkdB*, and *xpf* mutants.

### Effects of null mutations in genes involved in DNA repair and recombination on survival after replication arrest

In order to confirm the importance of the DNA repair and recombination genes identified in the Tn-seq screen, we made null mutations in six of the loci identified (*polA*, *recU*, *recN*, *recJ*, *walJ*, and *addAB*; note that *addB* and *addA* comprise an operon and for all experiments except where indicated, both genes were deleted) and tested their effects on survival after HPUra-mediated replication fork arrest. We treated wild-type and mutant cells with HPUra for 3 h and then removed HPUra and measured the number of viable cells remaining. Approximately 4.5% of wild-type cells survived this prolonged replication arrest (Fig 1B). In contrast, only 0.5–1% of *polA*, *recU*, *recN*, and *addAB* mutant cells survived.

In contrast to the detrimental effects of the loss of *polA*, *recU*, *recN*, and *addAB* on surviving replication fork arrest, we found that loss of *recJ* promoted survival. In the *recJ* null mutant, approximately 45% of cells remained viable after 3 hr of replication arrest (Fig 1B). These results indicate that normally RecJ is detrimental to survival of replication arrest in the absence of DNA lesions. Together, these results confirm the initial Tn-seq data that indicated that these genes are important for promoting survival following replication fork arrest in the absence of DNA damage.

### Comparison of *recJ* and *addAB* mutants in response to replication arrest

Both RecJ and AddAB are involved in end-resection (Fig 1A), and both improve survival after treatment with DNA-damaging agents [17–20]. Therefore, it was somewhat surprising that a *recJ* null mutant had increased survival in response to replication fork arrest in the absence of externally induced chemical damage to DNA.

We confirmed that Δ*recJ* and Δ*addAB* mutants were more sensitive than wild-type cells to treatment with the DNA damaging agent mitomycin C (MMC). Approximately 1.5% of wild-type cells survived treatment with MMC (0.33 μg/ml) for 3 h (Fig 1C). In contrast, *addAB* and *recJ* mutants had 7 and 4-fold lower survival, respectively.

To verify that the effects of *recJ* and *addAB* on survival and recovery from HPUra were due to replication arrest, rather than some other possible effect, we tested their survival under conditions in which replication was arrested with a temperature-sensitive allele of the replicative DNA helicase (*dnaCts*) [21]. Inactivation of the replicative helicase mirrored the phenotypes observed during HPUra-mediated replication arrest (Fig 1C). We grew *dnaCts* (LSF176), *dnaCts* Δ*addAB* (LSF326), and *dnaCts* Δ*recJ* (LSF274) strains at a permissive temperature (30°C). Cells were then shifted to non-permissive temperature (49°C) to arrest replication elongation. After 4 h at 49°C, we measured the number of viable cells from each of the three strains. In the otherwise wild-type cells, prolonged replication arrest at the non-permissive temperature caused a decrease in cell viability to about 20% of the original population (Fig 1C). The loss of *addAB* exacerbated this effect, and only 4% of cells were viable. In contrast, the loss of *recJ* largely protected the cells from the outcome of replication arrest due to inactivation of the replicative helicase, and 65% of cells survived (Fig 1C). Together, our results indicate that *recJ* is detrimental to surviving replication fork arrest in the absence of DNA lesions, and confirm previous work [17, 19] demonstrating that *recJ* contributes to survival following chemical damage to DNA.

### Loss of RecA loader RecO or regulator RecF protects Δ*addAB* cells from arrest-induced death

End-resection by AddAB or RecJ produces the substrate for interaction with RecA. RecO is the main protein responsible for loading RecA onto the ssDNA substrate (Fig 1A), and RecF stabilizes the resulting RecA nucleofilaments [11]. Insertions in *recO* and *recF* were not recovered in our initial Tn-seq screen because they were depleted from the initial library prior to treatment with HPUra (S1 Table). We suspect that this depletion was because cells were not kept in the dark during library preparation and these mutants are probably sensitive to ambient UV light.

**Fig 2:**
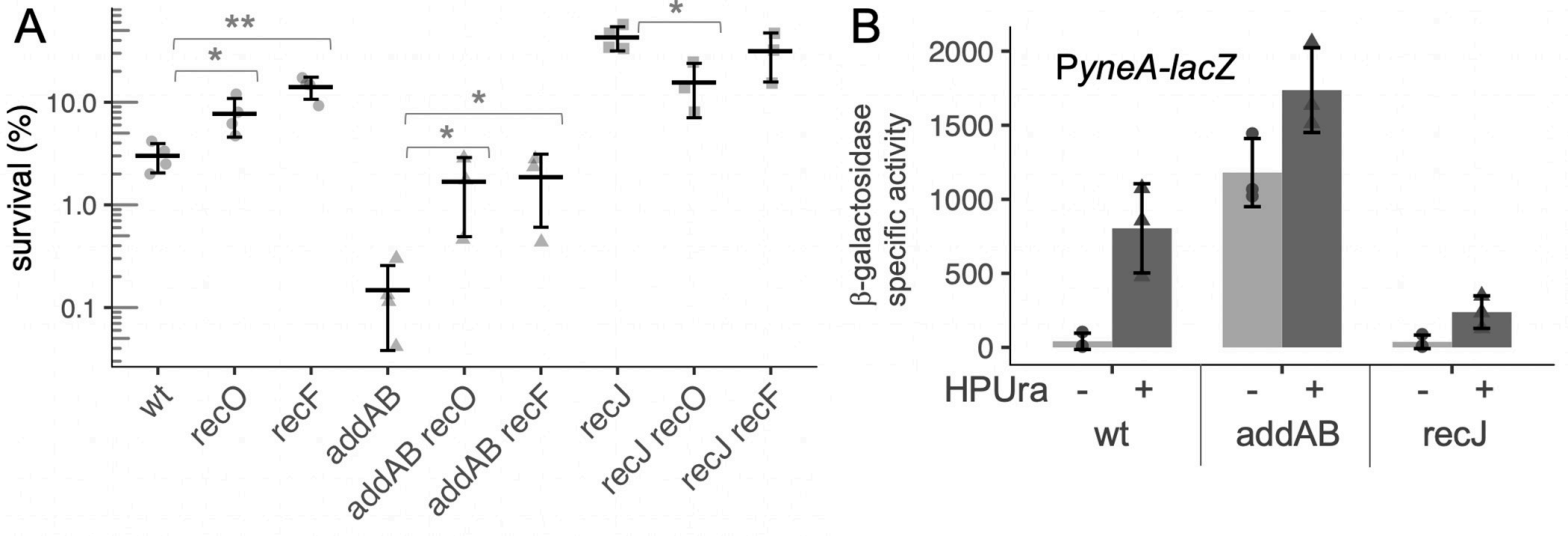
RecA loading is detrimental to survival during HPUra-induced replication arrest. **A.** Survival after HPUra treatment is greater in the absence of RecA loader and regulator (RecO, RecF). Fold change in survival after 3 h of arrest for wild type (JMA222), Δ*recO* (LSF814), Δ*recF* (LSF270), Δ*addAB* (LSF253), Δ*addAB* Δ*recO* (LSF817), Δ*addAB* Δ*recF* (LSF709), Δ*recJ* (LSF200), Δ*recJ* Δ*recO* (LSF815), Δ*recJ* Δ*recF* (LSF282). Significant difference in means (two-sample T-test): * P < 0.05; ** P < 0.01. **B.** RecA activity is increased in the absence of *addAB* and decreased in the absence of *recJ*. Transcription from the promoter of the SOS-inducible gene *yneA* is derepressed when RecA is active and serves as an indicator of RecA activity. Cultures of wild-type, Δ*recJ*, and Δ*addAB* strains containing *amyE*::*PyneA-lacZ* (LSF633, LSF634, and LSF635, respectively) were treated or not with HPUra. After 30 min, aliquots were collected for measuring ß-galactosidase activity. Results from three independent experiments are presented.

To test if *recO* and *recF* contribute to survival following replication arrest, we constructed *recO* and *recF* null mutants. We found that deletion of either *recO* or *recF* significantly increased cell survival following replication fork arrest (HPUra treatment) in comparison to wild type (Fig 2A). Both deletions suppressed the sensitivity of Δ*addAB* mutants to HPUra (Fig 2A, see *addAB recO* and *addAB recF*), indicating that loading RecA was at least partially responsible for decreased survival of the Δ*addAB* mutant.

In contrast to effects on the *addAB* mutant, deletion of *recF* had little if any effect on the sensitivity of the Δ*recJ* mutant to HPUra, indicating that RecF and RecJ likely affect the same process–formation of stable RecA filaments (Fig 2A).

### The SOS response is reduced in the absence of *recJ* and increased in the absence of *addAB*

We postulated that if the increased survival of Δ*recJ* is a consequence of lower stability of RecA filaments, then Δ*recJ* might have lower RecA activity. Once assembled onto ssDNA, RecA induces autocleavage of the SOS repressor LexA, allowing expression of many genes involved in the response to genotoxic stress [22–24]. Consequently, expression of genes repressed by LexA can be used as a proxy for the levels of activated RecA. *yneA* encodes an inhibitor of cell division [25], and is repressed by LexA and de-repressed in response to DNA damage and replication fork arrest [26].

We used a fusion of the promoter region of *yneA* to *lacZ* (P*yneA-lacZ*) and measured expression in the mutants in the presence and absence of HPUra. For wild-type cells in defined minimal medium, ß-galactosidase activity from the P*yneA-lacZ* fusion increased approximately 20-fold after 30 min of replication arrest (Fig 2B). In the Δ*recJ* mutant, there was a basal level of activity similar to that in wild type, but only an approximately 6-fold increase after addition of HPUra, indicating that *recJ* plays an important role in activation of RecA. The absence of *addAB* led to very high P*yneA-lacZ* activity during exponential growth (Fig 2B), indicating that there is an abundance of active RecA in these cells.

### Lysogenic phages are not responsible for the effects of *recJ* and *addAB* on survival after replication arrest

It seemed plausible that the impact of *recJ* and *addAB* on survival following replication stress could be due to different levels of phage induction, as both mutations affect the activity of RecA. The *B*. *subtilis* strains used in the experiments described above have two resident lysogenic phages, both of which undergo RecA-dependent activation after genotoxic stress: SPß and the defective phage PBSX [16, 26–28]. Both encode toxins and autolysins which, when expressed, can cause cell death. We found that insertions in two genes from SPß (*yoyI*, *yonH*) and three genes involved in the early steps of PBSX induction (*xpf*, *xkdBC*) were overrepresented in the screen, indicating that loss of these genes helped cells survive arrest of replication elongation (Table 1).

**Fig 3:**
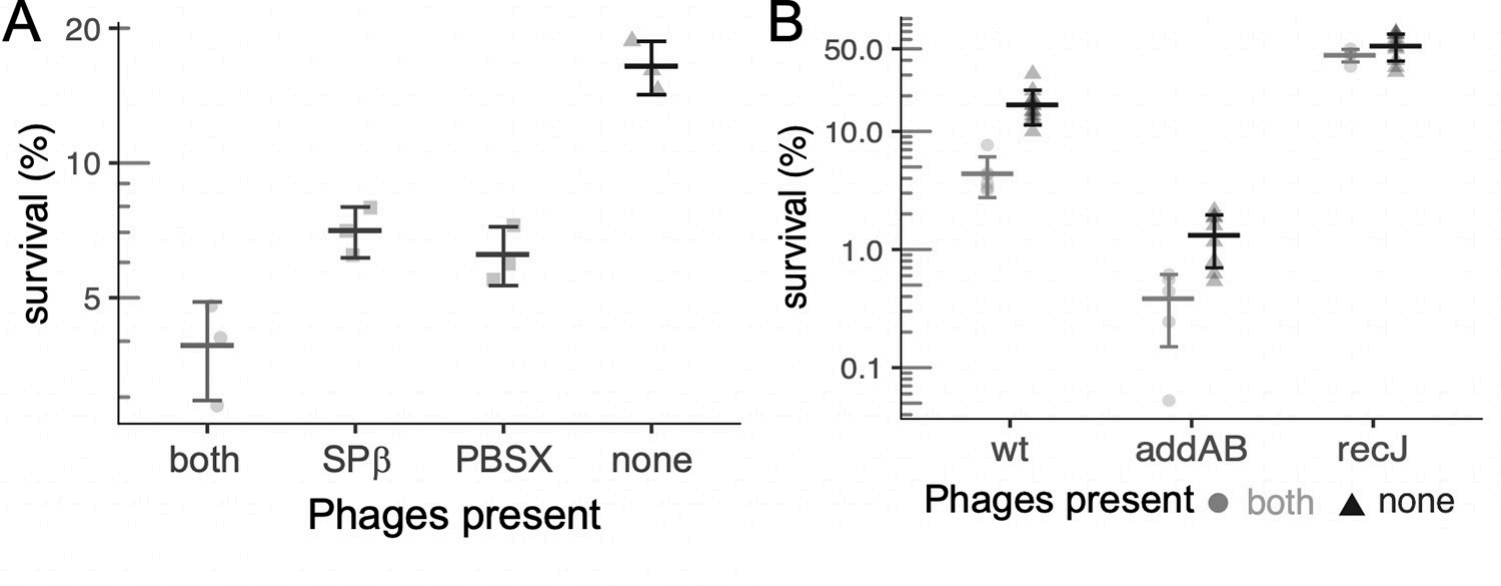
Effect of the resident lysogenic phages SPß and PBSX on survival after replication arrest. **A.** The lysogenic phages SPß and PBSX (defective) negatively affect cell survival after replication arrest (3 h HPUra treatment). Percent survival of strains JMA222 (which has both phages), LSF204 (only SPß; ΔPBSX), LSF203 (only PBSX; ΔSPß), and LSF225 (no phage; ΔPBSX, ΔSPß). **B.** *recJ* and *addAB* affect survival following replication arrest, even in cells without SPß and PBSX. Survival after 3 h of replication arrest for strains lacking both phage is plotted as dark gray triangles with black error bars; wt (LSF225), Δ*addAB* (LSF254), and Δ*recJ* (LSF231). Data from corresponding strains with both phages (JMA222, LSF253, and LSF200, respectively), plotted as light gray circles with gray error bars, are shown for comparison, and are the same as presented in Fig 1B. Statistically different means, by two-tailed t-test: * P < 0.05; ** P < 0.001; *** P < 0.0001).

We measured survival of otherwise wild-type cells missing either PBSX, SPß, or both following replication arrest with HPUra. In these experiments, the survival of wild-type cells (containing both lysogenic phages) was 3.5% (Fig 3A). Loss of either phage increased survival to approximately 7%, and loss of both phages increased survival to 15–20% (Fig 3A).

Although the presence of SPß and PBSX contributed to cell death following replication arrest, we found that *addAB* and *recJ* affected survival following replication arrest even in cells missing both PBSX and SPß. In strains cured of phages, loss of *addAB* caused a 10-fold drop in survival, indicating that this mutant was still more sensitive to replication arrest than wild-type cells (Fig 3B). This effect was comparable to that caused by loss of *addAB* in strains with both phages (~8-fold). The absence of *recJ* significantly increased the survival of cells after treatment with HPUra to ~50%, regardless of the presence or absence of PBSX and SPß. The increase in survival of the phage null mutant caused by loss of *recJ* was approximately 3-fold (Fig 3B). Together, these results indicate that the effects of *addAB* and *recJ* on survival are largely independent of killing by SPß or PBSX.

### End-resection is needed for survival following replication fork arrest, but RecA is not

We sought to determine how strains lacking *recJ* and *addAB-*encoded nucleases involved in end resection or lacking *recA* behaved following arrest of replication forks with HPUra. RecA is essential for survival following treatment with DNA damaging agents. Due to the inability to efficiently load RecA in the absence of the nucleases AddAB and RecJ, a double Δ*addAB* Δ*recJ* mutant has a similar extreme sensitivity to DNA damaging agents as Δ*recA* [17, 19, 29].

**Fig 4:**
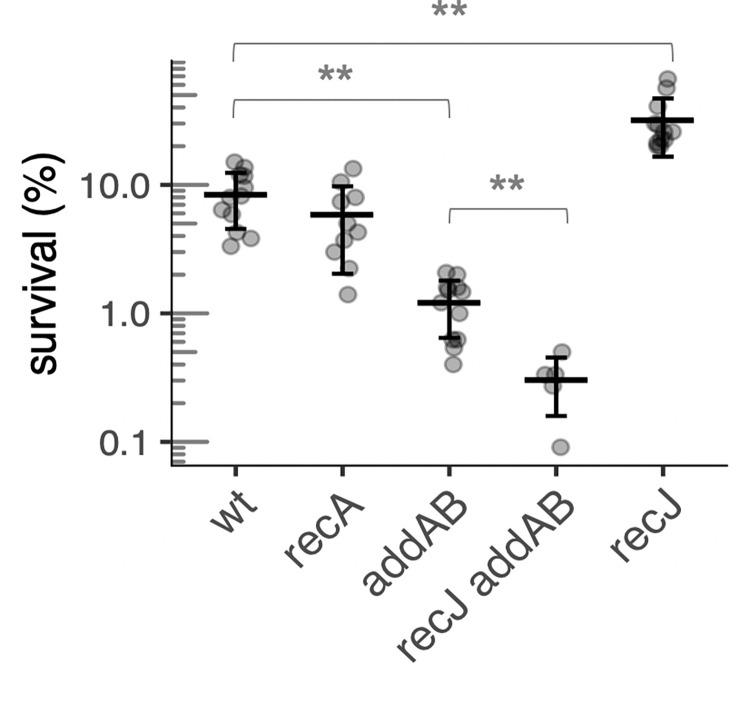
Effect of RecA and end-resection on survival of HPUra-induced replication arrest, in the absence of phages. Percent survival after 3 h of replication arrest for wild-type (LSF225), and *recA* (LSF658), *addAB* (LSF254), *recJ addAB* (LSF648), and *recJ* (LSF231) null mutants. ** Statistically different means, by two-tailed t-test (P < 0.005).

The survival following replication arrest of a strain missing both *addAB* and *recJ* was 0.3% (Fig 4). This is significantly worse than the effect caused by Δ*addAB* and indicates that the loss of both end-resection pathways is synergistic and that end-resection is needed for survival in response to replication arrest in the absence of chemical damage to the DNA.

In contrast, *recA* did not detectably affect survival following replication arrest. Survival of the *recA* null mutant was similar to that of otherwise wild-type cells, but lower than the *recJ* mutant (Fig 4). Based on these results, we conclude that end-resection mediated by either AddAB or RecJ is essential for recovery from replication fork arrest, but that RecA and RecA-mediated homologous recombination is not required.

It is formally possible that RecA is required in the absence of RecJ. For example, survival in the absence of RecJ depends on AddAB and this could be due to end processing by AddAB and subsequent assembly of RecA onto the ssDNA. If true, then survival of a *recJ recA* double mutant should be less than that of a *recJ* single mutant. We found that the increased survival conferred by the loss of *recJ* was not dependent on the presence of *recA* (Table 2), indicating that the AddAB pathway that is required for surviving replication arrest in the absence of RecJ was not dependent on RecA.

**Table 2:** *recA* is not needed for increased survival to replication stress in the absence of *recJ*.

Strain^1^	Survival relative to wild type^2^
Wild type (LSF225)	1.0 ± 0.5
*recJ* (LSF231)	3.8 ± 1.7
*recA* (LSF658)	1.0 ± 0.5
*recJ recA* (LSF659)	3.2 ± 1.4

^1^The indicated strains were grown to mid-exponential phase in defined minimal medium, treated with HPUra for 3 h, and the fraction of cells that survived were measured.

^2^Survival of each strain was determined and is presented relative to that of the wild type grown and treated in parallel. Data for each strain are averages and standard error of the mean from four independent matched experiments.

### RecA activity correlates with the accumulation of repair centers formed during HPUra-induced replication arrest

Since RecA itself was not required to survive HPUra-induced replication arrest, but mutants with lower RecA activity like Δ*recJ* and Δ*recF* had increased survival, we considered the possibility that the activity of RecJ and the formation of RecA filaments were interfering with the proper processing of the arrested replication forks. If true, then there should be greater accumulation of recombination intermediates and exposed DNA ends in strains with more loading of RecA. Upon formation of a double-strand break, the DNA ends are processed by end-resection allowing RecA to be loaded onto the ssDNA. It was previously believed that following chemical damage to DNA, RecN was recruited directly to dsDNA breaks before RecA loading [19, 30, 31]. However, recent evidence indicates that RecN is recruited to the already-processed DNA in a manner dependent on RecA [17]. Nonetheless, the formation of foci of RecN can be used as a proxy for DNA repair centers, likely as a result of a dsDNA break or gaps that have been processed to allow RecA to assemble into a filament [17, 19, 30, 31].

**Fig 5:**
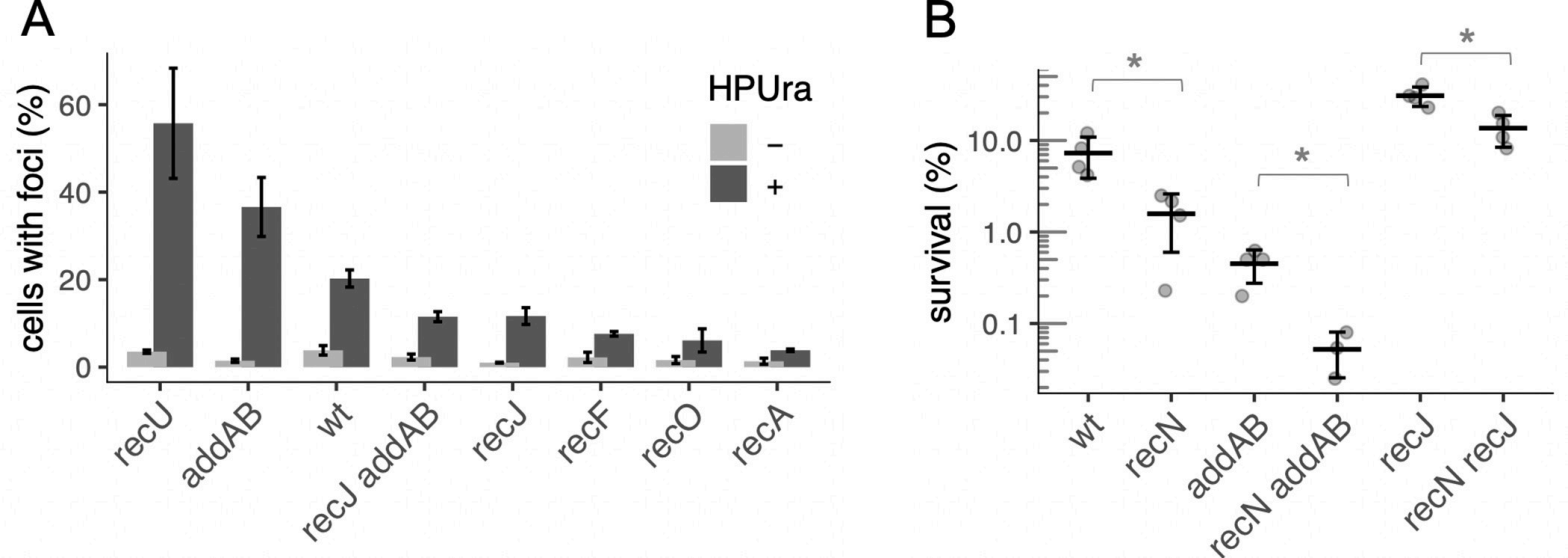
Accumulation of exposed DNA ends during replication arrest by HPUra. **A.** Repair centers in response to exposed DNA ends were visualized by fluorescence microscopy of cells expressing the RecN-mNeongreen fusion. Cell membranes were stained with FM 4–64 and DNA was stained with DAPI. Wild-type (LSF708), Δ*recU* (LSF740), Δ*addA* (LSF650), Δ*recO* (LSF818), Δ*recJ* (LSF649), Δ*recF* (LSF816), Δ*recJ* Δ*addA* (LSF706), and Δ*recA* (LSF756) cells were analyzed without (gray) or 30 min after treatment with HPUra (black). At least 800 cells from two biological replicates were analyzed from each strain for the presence of RecN-mNeongreen foci. The means and standard errors are presented. **B.** Loss of *recN* causes a phenotype in cells with or without *addAB* or *recJ*. Percent survival of wild type (LSF225), Δ*recN* (LSF536), Δ*addAB* (LSF254), Δ*recN* Δ*addA* (LSF652), Δ*recJ* (LSF231), and Δ*recN* Δ*recJ* (LSF651) are plotted. *Statistically different means according to two-tailed t-test: * P < 0.05.

RecN with a C-terminal tag has been shown to be functional in DNA repair and was used previously to monitor repair centers formed following DNA damage [17, 19, 30]. We constructed strains in which the native *recN* was replaced by *recN*-*mNeongreen*, and monitored formation of foci to determine the frequency of repair centers arising from DNA damage-independent replication arrest. Different mutants containing the fusion were treated with HPUra and scored by the presence of foci after 30 min of replication arrest (Fig 5A). In wild-type cells growing exponentially, cells with RecN-mNeongreen foci were uncommon (< 2% cells). Thirty minutes after replication arrest, approximately 20% of wild-type cells had at least one focus of RecN-mNeongreen (Fig 5A).

Effects of mutations on the formation of RecN-mNeongreen foci were significant. In the absence of *recU* or *addA*, 56% and 37% of cells, respectively, had at least one focus of RecN-mNeongreen (Fig 5A). In contrast, only ~12% of Δ*recJ* cells contained foci and a double Δ*recJ* Δ*addA* mutant behaved similarly to Δ*recJ* (Fig 5A). Finally, deleting *recA* or *recO* or *recF*, the genes encoding the RecA-loader and stabilizer, greatly reduced the number of foci of RecN-mNeongreen (Fig 5A). This indicates that association of RecN to the DNA damage centers is dependent on RecA during damage-independent replication arrest and is consistent with similar findings after treatment with agents that induce DNA damage [17].

These results support the model that RecJ and the recombinase loader and regulator impede the proper processing of the replication fork via excessive formation of RecA filaments in the absence of chemical damage to DNA, and indicate that the RecJ pathway is the major source of RecA assembly following HPUra-induced replication arrest. The neutral effect of *recA* in survival (Fig 4) may be a combination of increased survival due to lack of loading on DNA processed by RecJ and a decrease in RecA-mediated processes for restart. In addition, there are likely to be RecA-independent processes for replication restart (see Discussion).

### RecN is important for survival if RecA is assembled onto DNA

Our results indicate that there are breaks or gaps in dsDNA that lead to DNA processing and RecA loading in wild-type cells following replication fork arrest. Furthermore, we found that the loss of *recN* caused a decrease in survival in otherwise wild-type cells following replication fork arrest (Table 1 and Fig 5B). In the absence of *addAB*, there was a further decrease in survival upon loss of *recN* (Fig 5B), consistent with the results indicating that RecA is more active and there are more dsDNA breaks in the absence of *addAB* than in otherwise wild-type cells. In addition, loss of *recN* also decreased survival of the *recJ* mutant (Fig 5B), indicating that RecN is beneficial regardless of which pathway (AddAB or RecJ) is used to process DNA ends.

## Discussion

The replisome pauses or collapses not only when it encounters damaged DNA, but also when roadblocks, such as DNA binding proteins, are encountered, or when the replisome itself is inhibited or damaged. We specifically focused on how cells recover from replication arrest by replisome inhibition, in the absence of chemical damage to DNA. It is well known that RecA activity and both the RecJ and AddAB end-resection pathways are critically important for surviving exposure to DNA damage-inducing agents [32–34]. In contrast, we found a different scenario when there is no DNA damage and a replisome component is inhibited (chemically or using a thermosensitive allele). In this situation, end-resection was necessary, but the RecJ pathway and high RecA activity were detrimental, and the AddAB pathway was beneficial. Our results show that different types of genotoxic stresses require different levels of RecA activity, which are obtained by the AddAB vs. RecJ pathways, and that the choice of pathway affects survival.

### The consequences of using each end-resection pathway depend on the kind of replication arrest

**Fig 6:**
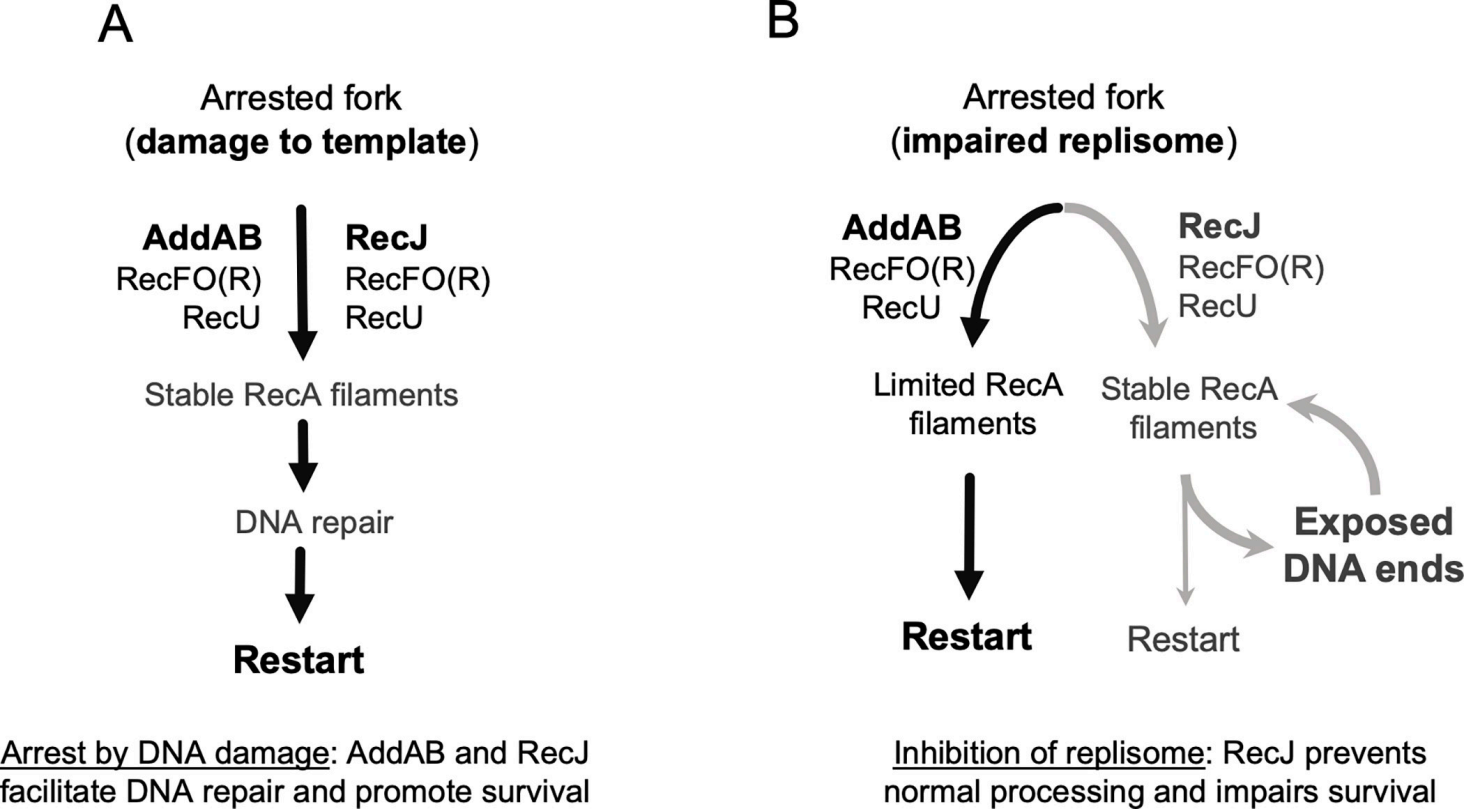
The role of end-resection pathways AddAB and RecJ on survival outcomes after replication arrest. **A.** When replication arrest is caused by lesions on the DNA template or other genotoxic stresses requiring RecA activity, both end-resection pathways work together, generating stable RecA filaments that delay restart and support DNA repair. **B.** If replication arrest is caused by the inhibition of replisome components or other stresses that do not require RecA, AddAB favors a pathway (black arrows) with limited RecA filaments that promotes survival. In this situation, RecJ and the RecA loader RecO and regulator RecF impair survival by promoting a pathway (gray arrows) with high RecA activity. This could prevent proper processing of the fork, exposing DNA ends and creating a futile circle.

As depicted in Fig 6A, when replication is blocked, both end-resection pathways work together to process the collapsed replication fork. After fork regression, which involves reannealing of the template DNA and annealing of the two daughter strands, AddAB can unwind and degrade the daughter strands until it reaches a Chi site, after which it begins generating a 3’ ssDNA tail [10]. RecJ can target the collapsed fork directly, or an already resected fork [35], probably extending the ssDNA substrate generated by AddAB and leading to longer RecA nucleofilaments. RecFO(R) then ensures RecA nucleofilament loading and stability [11]. In this case, the role of RecA is not to promote recombination, but rather to stabilize the DNA at the fork until the DNA damage has been repaired [32, 33]. Our results indicate that when replication stops because a replisome component is inhibited, AddAB activity supports optimal recovery (black arrows in the left side of Fig 6B) and after fork regression, AddAB generates less substrate for RecA loading than does RecJ.

Our results indicate that RecJ and the loader RecO (and regulator RecF) negatively affect the process of fork repair by enhancing RecA activity and preventing fork regression (gray arrows by the right side of Fig 6B). RecJ can target a range of DNA substrates [9, 36]. In addition to competing with AddAB for access to an already regressed fork, it can degrade a daughter strand in the collapsed fork, which would prevent fork regression and the generation of the blunt ends AddAB requires. Also, RecJ and RecO localize to the replication fork via interaction with SSB [37], and therefore have immediate access to the replication fork, whereas AddAB does not. In the absence of DNA damage, the resulting stable RecA filaments that help promote survival in other conditions may expose DNA ends that unnecessarily delay fork processing. Any DNA breaks or exposed ends arising during this step would need to go through an additional round of end-resection and RecA loading, feeding the detrimental pathway.

### Different types of genotoxic stresses require different levels and duration of RecA activity

The different effects that RecA has on survival, depending on the kind of replication arrest, could be due to the complexity of the resulting collapsed fork and duration of RecA activity necessary for restart. Treatment with MMC, for example, impedes replication due to template lesions and “dirty” DNA breaks, *i*.*e*. ones that cannot be ligated because they lack a 3’ hydroxyl or 5’ phosphate. The recovery from this kind of arrest can take up to 3 h in *B*. *subtilis* and relies on high levels of RecA activity [29, 30]. The repair of a “clean”, ligatable single break in the chromosome, however, requires very transient RecA filaments and takes less than 20 min, at least in *E*. *coli* [38].

When a DNA-binding protein acting as a roadblock arrests replication, *E*. *coli* cells are able to process these forks and recover normally, regardless of the presence of *recA* [39]. In this case, there are no chemical lesions to be repaired or complex structures to be disassembled at the fork before processing. Likewise, our data indicate that the collapsed forks resulting from the inhibition of a replisome component (PolC by HPUra treatment or arrest of the replicative helicase DnaC using at temperature sensitive mutant) readily undergo fork regression and processing. In this scenario, we found that the excess RecA activity that results from RecJ and the recombinase loader and regulator is not only unnecessary but also detrimental to survival.

Replication-transcription conflicts also cause replication arrest (reviewed in [3, 40]). The importance of RecA in replication restart at these sites is unclear. One report found that RecA was not required in *E*. *coli* [41], but another found that RecA was needed in *B*. *subtilis* [42]. The fact that RecA is required in *B*. *subtilis* for resolving head-on replication-transcription conflicts, but not for recovery from replication arrest caused by HPUra may be due to the different structures that are formed after each of these treatments. HPUra treatment has been shown to leave many of the replisome components largely intact, at least initially [43–46], whereas replication-transcription conflicts likely cause replisome disassembly [47], and this may predispose arrested forks to different restart pathways.

### RecJ leads to increased RecA loading and DNA repair centers and increased cell death when fork repair does not require RecA activity

We hypothesize that during HPUra-induced arrest in a wild-type cell, the stable RecA nucleofilaments favored by RecJ prevent optimal repair of the collapsed fork by unnecessarily delaying restart, perhaps by inhibiting the ability of PriA to function or increasing the frequency of double-strand breaks, or both. We noted a positive correlation between the predicted levels of RecA activity in different mutants following HPUra treatment and the accumulation of DNA repair centers (increased RecN-mNeongreen foci; Fig 5A). These are likely a result of increased DNA breaks or gaps that have undergone end-resection and RecA loading, followed by RecN recruitment [17, 48]. Stable RecJ-dependent RecA filaments may factor into the propensity to cause breaks in these long stretches of coated ssDNA, leading to more DNA repair centers. Similar to what is observed for repair in the context of DNA damage, we found that deletion of *recN* decreased survival of HPUra treatment in wild type, Δ*addAB*, and Δ*recJ* mutants, indicating that under all of these conditions, recruitment of RecN helps to repair the DNA, regardless of the level of activity of RecA. These two mechanisms–increased DNA repair centers (likely from increased dsDNA breaks or gaps) and unnecessarily stalled restart–presumably synergistically contribute to cell death during HPUra treatment.

Few studies have focused on the survival outcome of recombination mutants to replisome inhibition in the absence of external DNA damage. Nevertheless, there are some hints of a disadvantageous role of RecJ in situations where RecA is not required in *E*. *coli*, which could be explained by excessive loading of the recombinase. A strain lacking *recB* (analogous to Δ*addAB* in *B*. *subtilis*) can still process a fork after collapse due to a roadblock, but survival was low, while a strain lacking *recA* was indistinguishable from wild type [39]. Although a mechanism for these findings was not proposed, the results indicate that a pathway parallel to RecBCD (presumably RecJ/gap repair) may be processing the collapsed forks in a way that decreases viability. Toxicity caused by RecJ, RecFOR, and RecA also occurs in the absence of the helicases (DNA translocases) encoded by *rep* and *uvrD* in *E*. *coli* and *pcrA* in *B*. *subtilis*. This toxicity is apparently due to excess or improper loading of RecA at blocked replication forks and led to the understanding that the helicases normally help remove or clear RecA to ensure proper replication restart [49–51]. These examples highlight the importance of controlling RecA loading and how improper or excessive loading can be detrimental to the cell.

RecJ homologs are widespread from bacteria to eukaryotes [52]. It is not known if the detrimental nature of RecJ and excessive loading of RecA in response to replication arrest is widespread, but the results from *E*. *coli* discussed above, and the conserved nature of RecJ and RecA indicate that this might be the case.

### Possible mechanisms of surviving replication fork arrest in the absence of RecA

Our data indicate that although RecA is dispensable, and at times detrimental, to the repair of replication arrest in absence of external DNA damage, end-resection is still required for survival. This indicates that formation of RecA-dependent Holliday junctions is not required for survival, and that the end-resection has a purpose other than loading of RecA. In *E*. *coli*, replication fork reversal can be accomplished independently of RecA, using RecBCD (analogous to AddAB in *B*. *subitilis*) and RuvAB, and has been observed in situations of stalled forks in the absence of chemical damage to DNA, including fork stalling caused by mutations in the DNA polymerase holoenzyme (reviewed in [2]), conflicts caused by DNA binding proteins [39], or head-on collisions with RNA polymerase [41]. A similar mechanism may occur in the absence of RecA after HPUra treatment, however it is also possible there are other RecA-independent mechanisms in *B*. *subtilis*, perhaps including non-homologous end-joining [53] or a role for DNA polymerase I [54, 55]. Additionally, breaks that occur at the replication fork resulting in damage to only one copy of the chromosome would yield one viable cell, rather than two, but would not be lethal nor require recombination. Indeed, in *E*. *coli*, in the absence of RecA, damaged chromosomes are degraded by RecBCD and can result in cells containing an odd number of chromosomes due to selective degradation of the damaged chromosomes [56].

### Bacteria use all pathways for repair, even when it hinders proper recovery

Each end-resection mechanism seems adapted to a kind of genotoxic stress in *B*. *subtilis*: AddAB for double-strand breaks from damage or fork collapse, and RecJ for gaps from DNA repair. However, our data highlighted that bacteria may use an unfavorable (“wrong”) pathway for repair. RecJ commits repair of some DNA damage-independent collapsed forks to a pathway that decreases viability, and it probably competes with AddAB for blunt DNA ends. RecJ and stable RecA loading only promote survival during DNA repair, but our data indicate that they are also used during replication stresses that do not require RecA. Ultimately, this indicates that cells do not have an effective mechanism to discriminate between these different types of broken forks, potentially leading to increased cell death in some, but enabling robust repair and restart in other circumstances.

## Materials and methods

### Bacterial strains

Unless otherwise indicated, all *B*. *subtilis* strains used in this work derive from JMA222 [57], a version of JH642 [58] cured of the integrative and conjugative element ICE*Bs1*. Strain genotypes and references for previously described strains are listed in Table 3. Previously described alleles were introduced into JMA222 by transforming naturally competent cells with genomic DNA from the relevant strain. These alleles include: *dnaC30(ts)-mls* [21], *recU*::*cat* [59], *recA260* [60], *walJ*::*erm* [61], *recF*::*spc* [62], *amyE*::P*yne-lacZ* cat [63], *ponA*::*spc* [64], and *addAB*::*kan* [65].

**Table 3:** *B*. *subtilis* strains used.

Strain	Relevant genotype^a^ [reference]
AG174 (a.k.a., JH642)	*pheA1 trpC2* [58, 66]
BKE40370	*walJ*::*erm* (168 background) [61]
CMJ293	*ponA*::*spc*
CMJ374	*cwlO*::*spc*
GP891 (a.k.a., LSF625)	*recU*::*cat trpC2* ICE*Bs1* (168 background) [59]
JMA222	*pheA1 trpC2* ICE*Bs1*^0^ [57]
JRL131	Δ*oppA* (AG174 background) [67]
JRL189	Δ*oppB* (AG174 background) [67]
LSF20	*speE*::*spc*
LSF41	*polA*::*cat* (a.k.a., AB41)
LSF176	*dnaC30*(ts)-*mls*
LSF197	ΔSPß::*lox*-*kan*-*lox*
LSF198	ΔPBSX::*lox*-*kan*-*lox*
LSF200	*recJ*::*spc*
LSF201	*Δ*PBSX::*lox-kan-lox* (PY79 strain background) [68]
LSF203	ΔSPß::*lox*
LSF204	ΔPBSX::*lox*
LSF225	ΔSPß::*lox* ΔPBSX::*lox*
LSF231	ΔSPß::*lox* ΔPBSX::*lox recJ*::*spc*
LSF233	ICE*Bs1*^0^ *hrcA*::*cat*
LSF253	*addBA*::*kan*
LSF254	ΔSPß::*lox* ΔPBSX::*lox addBA*::*kan*
LSF270	*recF*::*spc* (a.k.a., CAL1614)
LSF274	*dnaC30*(ts) *recJ*::*spc*
LSF282	*recJ*::*cat recF*::*spc*
LSF284	*recN*::[*recN*-9aa-*mNeongreen kan*]; referred to as *recN*-*mng kan*
LSF298	*recN*::*kan*
LSF326	*dnaC30*(ts) *addBA*::*kan*
LSF444	SPß::*lox* PBSX::*lox ycgE*::*spc*
LSF536	ΔSPß::*lox* ΔPBSX::*lox recN*::*kan*
LSF633	*amyE*::[P*yneA-lacZ cat*]
LSF634	*amyE*::[P*yneA-lacZ cat*] *recJ*::*spc*
LSF635	*amyE*::[P*yneA-lacZ cat*] *addBA*::*kan*
LSF648	ΔSPß::*lox* ΔPBSX::*lox recJ*::*spc addBA*::*kan*
LSF649	ΔSPß::*lox* ΔPBSX::*lox recN*-*mng kan recJ*::*spc*
LSF650	ΔSPß::*lox* ΔPBSX::*lox recN*-*mng kan addA*::*spc*
LSF651	ΔSPß::*lox* ΔPBSX::*lox recN*::*kan recJ*::*spc*
LSF652	ΔSPß::*lox* ΔPBSX::*lox recN*::*kan addA*::*spc*
LSF658	ΔSPß::*lox* ΔPBSX::*lox recA260* (*mls*, *cat*)
LSF659	ΔSPẞ::*lox* ΔPBSX::*lox recJ::spc recA260* (*mls, cat*)
LSF706	ΔSPß::*lox* ΔPBSX::*lox recN*-*mng kan recJ*::*cat addA*::*spc*
LSF708	ΔSPß::*lox* ΔPBSX::*lox recN*-*mng kan*
LSF709	*addBA*::*kan recF*::*spc*
LSF740	ΔSPß::*lox* ΔPBSX::*lox recN*-*mng kan recU*::*cat*
LSF756	ΔSPß::*lox* ΔPBSX::*lox recN*-*mng kan recA260* (*mls*, *cat*)
LSF814	*recO*::*cat*
LSF815	*recO*::*cat recJ*::*spc*
LSF816	ΔSPß::*lox* ΔPBSX::*lox recN*-*mng kan recF*::*spc*
LSF817	*recO*::*cat addBA*::*kan*
LSF818	ΔSPß::*lox* ΔPBSX::*lox recN*-*mng kan recO*::*cat*

^a^ Unless otherwise indicated, strains are derived from JMA222, and the *trpC2*, *pheA1*, and ICE*Bs1*^0^ alleles are not shown.

Introduction of new alleles was done by transforming cells with linear DNA products generated either by traditional cloning or by isothermal assembly [69]. Insertion-deletion constructs contained antibiotic resistance cassettes typically flanked by 800–1000 bp of genomic sequences upstream and downstream of region to be deleted. These alleles include *addA*::*spc*, *recJ*::*spc*, *recN*::*kan*, *recO*::*cat*, *recF*::*spc*, *ycgE*::*spc*, *hrcA*::*cat*, *polA*::*cat* and *cwlO*::*spc*.

To generate deletions of SPß (LSF203) and PBSX (LSF204), each was substituted by a kanamycin resistance gene flanked by *loxP* sites, generating strains LSF197 (ΔSPß::*lox*-*kan*-*lox*) and LSF198 (ΔPBSX::*lox*-*kan*-*lox*). The cassettes were removed by the Cre recombinase (from bacteriophage P1) using the *cre* expression vector pDR244 as previously described [70], leaving a 75 bp insertion (’scar’ containing a single *lox* site). A strain devoid of both phages (LSF225) resulted from transforming LSF203 (ΔSPß::*lox*) with genomic DNA from LSF201 (a ΔPBSX::*lox*-*kan*-*lox* strain), and recombining the antibiotic cassette out. The *Δ*PBSX allele also removes *spoIISABC* (toxin-antitoxin-antitoxin) which is just downstream of PBSX but is not normally thought to be part of it [71].

To visualize DNA repair centers, we constructed a strain with a *recN*-*mNeongreen* (*recN*-*mng*) fusion, inserted in the native locus such that the fusion is the only *recN* allele. The full linear DNA product contained the 800 bp at the 3’ end of *recN*, an in-frame linker (5’- CTCGAGGGATCTGGCCAAGGAAGCGGC-3’; encoding 9 amino acids), the mNeongreen coding sequence (a gift from Ethan Garner), a kanamycin resistance cassette, and 800 bp genomic sequence downstream of the *recN* stop codon. Transformation of this construct into JMA222 generated strain LSF284 that contains *recN*::[*recN*-9aa-*mNeongreen kan*]; referred to as *recN*-*mng kan*. The *recN*-*mng kan* allele from LSF284 was then introduced into various strains, including LSF225 (generating LSF708).

### Media and antibiotics

All experiments were performed in defined minimal medium containing 50 mM MOPS (S7_50_) and supplemented with 1% glucose, 0.1% glutamate, 40 μg/ml phenylalanine and 40 μg/ml tryptophan [72]. When required, the following concentration of antibiotics were used: 5 mg/ml chloramphenicol, 100 μg/ml spectinomycin, 5 μg/ml kanamycin, and 0.5μg/ml erthyromycin plus 12.5 μg/ml lincomycin to select for macrolide-lincosamide-streptogramin B (MLS) resistance. Serial dilutions of cultures for spot-plating were made in Spizizen minimal salts medium [73].

### Viability assays

*B*. *subtilis* strains were streaked from -80°C freezer stocks on LB plates and grown overnight at 37°C. Single colonies were transferred to S7_50_, and dilutions of those were grown overnight with vigorous shaking at 37°C. Starter cultures between OD_600_ 0.05–0.5 were diluted to OD_600_ 0.025, grown for at least three generations, and adjusted to OD_600_ 0.1. *recA* mutants were grown in the dark (Δ*recA* mutants, and many other mutants altered in the DNA damage response, are sensitive to ambient UV light). HPUra (6-(p-hydroxyphenylazo)-uracil; [11]), 38 μg/ml, or mitomycin C (Sigma-Aldrich), 0.33 μg/ml, was added as indicated, and cultures were kept at 37°C with shaking for 3 h. Viability was assessed before and at various times after addition of HPUra or MMC by making 10-fold serial dilutions from 100 μl of cultures in a 96-well plate and spotting 10 μl on LB plates, in triplicate. Results are in percentage of colony forming units present after 3 h treatment in relation to immediately before addition of HPUra or MMC.

Temperature sensitive mutants with the *dnaCts* (replicative helicase) allele [21] were grown at 30°C. At OD_600_ 0.1, the cultures were shifted to 49°C, the non-permissive temperature, to arrest DNA replication. Dilutions of the cultures were plated before and every hour after the shift to assess survival. Results are percentages of colony forming units (at 30°C) present after 4 h in relation to immediately before the temperature shift.

Statistical comparison between different groups was performed in R, using the varequal and t.test functions. Two-tailed, paired t-tests were computed using a pooled estimate of variances for similarly distributed samples or were estimated separately for both groups and the Welch modification to the degrees of freedom was used.

### Tn-seq screen

The transposon insertion library used in this work has been described in detail [15]. The library contains ~1–2 x 10^5^ unique transposon insertions in strain JMA222.

An aliquot of the transposon insertion library was diluted in minimal medium, and grown from an OD_600_ of 0.02 to 0.3 in defined minimal medium at 37°C. The culture was then split and HPUra (38 μg/ml) was added to half to arrest replication, and the other half was untreated. After 1 h at 37°C, cells from both cultures were harvested by centrifugation, washed, and resuspended in fresh minimal medium, diluting the cultures back to OD_600_ 0.15. They were allowed to recover for 4 h, and aliquots were harvested every hour during this time. DNA was prepared for sequencing as described previously [15], and sequencing was done using an Illumina HiSeq by the MIT BioMicro Center.

### Tn-seq data analysis

Tn-seq data were initially processed as described [15]. Sequences adjacent to the transposon were mapped to JMA222 genome using Bowtie2 [74]. The resulting files contained the number of reads per genomic coordinate in each sample. Our resulting mapped libraries had approximately 10^5^ independent insertions each, with an average of one insertion per 37 base pairs, and 19 insertions per non-essential gene.

Subsequent analyses and file manipulations were performed using custom-made R scripts. Any genomic position with less than 3 reads was discarded, to avoid potential noise due to rare insertions or misaligned reads. Then, we used inter-sample quantile normalization from the preprocessCore package (available at www.bioconductor.org) to ensure that different samples were comparable. Finally, we calculated the number of reads interrupting each gene in every sample–only insertions mapping to 5–95% internal sequence were considered since insertions in the extremities of essential genes are sometimes tolerated.

To assess enrichment or depletion of insertion mutants, we calculated the ratio between the number of reads in the treated and control samples. For proper analysis of Tn-seq, it is important that libraries being compared were expanded roughly the same number of generations before sequencing. Since replication and cell division in the treated library was arrested during 1 h (leaving them one generation “behind” the control libraries), we compared the HPUra-treated libraries with the controls harvested an hour earlier: the HPUra sample harvested after 4 h of recovery was compared to the control harvested after 3 h, and HPUra 2 h with control cells harvested after 1 h of recovery.

Finally, for a gene to be considered to affect survival in HPUra in relation to the control, it had to satisfy the following requisites: (i) be longer than 200 bp; (ii) have more than 5 insertions in the corresponding control library; (iii) have log2 fold change > 1 at 4 h; (iv) have an amplified change in read frequency over time. These criteria aimed to restrict the number of candidate genes and decrease false-positives. The importance of additional candidate genes of interest (*e*.*g*., *recO*, *recF*, and *recA*) that did not meet all of these criteria were evaluated using targeted gene disruptions.

### ß-Galactosidase activity assay

Cultures in mid-exponential phase growing in minimal medium were diluted to OD_600_ 0.1. One-milliliter aliquots were permeabilized with 15 μl toluene and stored at -20°C. For cells undergoing replication arrest, HPUra (38 μg/ml) was added for 30 min, 1.5 ml aliquots were centrifuged at 3000 x g for 2 min and cells were resuspended in 1.5 ml of fresh medium. One milliliter aliquots were permeabilized and frozen, and the remainder was used to measure OD_600_ to correct for cell recovery. ß-galactosidase specific activity was determined as previously described [75, 76].

### Fluorescence microscopy

We used RecN-mNeongreen as a marker to measure DNA repair centers. Cells containing this construct were grown in minimal media until OD_600_ ~ 0.1. Cells were either untreated or treated with HPUra (38 μg/ml) for 20 min. Aliquots were then added to a tube containing 4’,6’-diamidino-2-phenylindole (DAPI, 1 μg/ml final concentration) and FM4-64 (2.5 μg/ml), to stain nucleoids and membranes, respectively, incubated for 10 min at 37°C, and then prepared for imaging.

For imaging, cultures were concentrated roughly four-fold by centrifugation (2 min at 2000x g) and 2 μl cells were placed on a slice of 1.5% UltraPure agarose (Invitrogen) made with minimal medium. The agarose slice was placed, cells down, on standard coverslips and imaged on a Nikon Ti-E inverted microscope. Fluorescence was generated using excitation/emission of 500/535 nm for RecN-mNeongreen (1 ms exposure), 350/460 nm for DAPI-stained nucleoids (100 ms) and 510/630 for FM4-64-stained membranes (100 ms). Image processing was performed using Fiji [77], where foci were detected with the Laplacian of Gaussians (LoG) detector in the TrackMate plugin [78], with a 0.7 nm blob diameter and threshold between 10 and 20 (determined by the control samples).

## Supporting information

S1 Table Data from Tn-seq analysis with the number of insertions and read frequency of transposon insertions in each gene with (indicated as ’treated’) and without (indicated as ’control’) treatment with HPUra. Samples were collected and analyzed 2, 3, and 4 hours after treatment, or from parallel untreated cultures. The sequencing data used for these analyses have been deposited in the NCBI Gene Expression Omnibus [79] and are accessible through GEO Series accession number GSE221151.
(XLSX)

S1 Data Underlying raw data for experiments presented. The excel spreadsheet contains the underlying data for the experiments presented in each of the figures and Table 2.
(XLSX)
